# Highlights of the first ISCB Student Council Symposium in Africa 2015

**DOI:** 10.12688/f1000research.6877.1

**Published:** 2015-08-17

**Authors:** Yassine Souilmi, Imane Allali, Oussama Badad, Chinmay Kumar Dwibedi

**Affiliations:** 1 Department of Biology, Mohamed Vth University, Rabat, 110 066, Morocco; 2 Department of Biomedical Informatics, Harvard University, Cambridge, MA, 02138, USA; 3 Department of Clinical Microbiology, Umea University, Umea, 901 87, Sweden; 4 Swedish Defence Research Agency, Umea, 164 90, Sweden

**Keywords:** Computational biology, bioinformatics, Africa, student council, ISCB, symposium

## Abstract

This is a summary of the activities and scientific content of the first International Society for Computational Biology Student Council symposium in Africa. This meeting organized by the students for the students took place 8th of March 2015 in Dar Es Salaam, Tanzania.

## Background

Bioinformatics is a rising field in Africa. The US National Institutes of Health (NIH) and the British Wellcome Trust, conscious of its importance, have funded the creation of a continent-wide bioinformatics network that brings together researchers and from different countries and groups through the Heath Human Heredity in Africa (H3Africa) project (H3ABioNet is funded by NIH Common Fund Award/NHGRI Grant Number U41HG006941). This resulted in the creation of the pan-African Bioinformatics Network for H3AbioNet, a 32-node network based in 15 African countries with a primary mission to help connect and collaborate, in order to introduce bioinformatics into the life sciences, agronomic and health research in Africa.

The Regional Students Groups (RSGs) of Africa, the local International Society for Computational Biology (ISCB) Student Council (SC) chapters, has its mandate to network and support the next generation of computational biologists ^1, 2^.

In this favorable environment, we organized the first Student Council Symposium in Africa (SCS-Africa, website: http://scs-africa.iscbsc.org) as a student-focused meeting alongside the “ISCB Africa ASBCB 2015 conference”. SCS-Africa will take place every two years following the schedule of the main conference.

## SCS-Africa: first edition

The first edition of the SCS-Africa took place March 8th, 2015 in Dar Es Salaam, Tanzania. The meeting was held one day before the ISCB/ASBCB conference.

### Symposium objectives

The symposium intended to accomplish the three following objectives:

1. Provide an opportunity to young African researchers and students to present their work among a focused audience of their peers.
2. Foster contacts and create a network of students and young researchers in Africa.
3. Give the participants a chance to interact with international level speakers.

### Symposium format

The SCS-Africa is a half-day event. This first edition kicked off with a welcome note from the organizers and Dr. Manuel Corpas, the founder and inaugural chair of ISCB Student Council and elected Board of Directors for the Society since 2014. The scientific program consisted of two keynote addresses, six oral presentations and a short training session. The symposium was concluded by a social hour and award winner announcement.

Dr. Manuel Corpas (TGAC, Norwich, UK) and Dr. Akinleye Callistus Adewale (LAUTECH Teaching Hospital, Osogbo, Nigeria) gave keynote presentations. In addition Yassine Souilmi, meeting Chair, RSG Vice Chair for Africa and Fulbright Fellow at Harvard Medical School, gave a short introductory session on cloud computing for computational biology. SCS-Africa abstract submission went through a peer-review process by independent reviewers. Seven abstracts were accepted for oral presentations. All abstracts are available online in the SCS-Africa 2015 book of abstracts ^3^.

The symposium attracted undergraduate, graduate students and young researchers from different fields including biologists, geneticists, physicists, mathematicians, statisticians, and software developers that either work with biomedical or biological global data, and who had an interest in bioinformatics and computational biology.

## Symposium’s highlights

### Welcome address: first edition of the SCS-Africa

The formal kickoff of the first edition of the SCS-Africa started with a welcome address putting the delegates in the context of the meeting, and was also marked by the presence of Dr. Corpas who greeted the delegates and followed with a keynote address where he presented the perspectives of the meeting.

### Keynotes

Dr. Manuel Corpas’ keynote presentation was a followup of the welcome address. Dr. Corpas displayed statistics and evidences of the high importance of networking and volunteering work and its impacts on young scientist careers, especially at the student level. Dr. Corpas illustrated with his personal experience as Founding Chair of the SC and its positive impacts on his own career.

Dr. Akinleye Callistus Adewale’s keynote presentation offered a key overview on the challenges being faced by bioinformaticians in the medical field in general and in Africa specifically. Dr. Adewale presented a qualitative study from the Nigerian Teaching Hospital.

### Student presentations

Olaitan Awe gave the first oral presentation; he introduced “A Comparative Analysis of the Genetic Relationships Between the Pathogens of Ebola Hemorrhagic Fever, Marburg Virus, HIV, Hepatitis A, Hepatitis B, Hepatitis C, Hepatitis D, and Hepatitis E”. In his work, he highlighted the genetics of the Ebola genome with the genome of seven other viruses to identify points of significant similarities and disparities. This comparative analysis can help to provide innovative methods in understanding the Ebola menace ^4^.

Soyemi Jumoke introduced “Computational Pharmacology Modelling: The RAFAGene Tuberculosis Pharmacokinetic as a case study”. He is interested in the RAFAgene drug trial for Tuberculosis in order to study the Adverse Drug Reaction (ADR) caused by the use of medicine, which pose risk from future exposure and requires prevention or specific treatment. He will be using a machine-learning approach by combining phenotypic, intrinsic and topological features of drugs with chemical and biological properties of drug as well as protein target and pathway information ^5^.

Mohamed Alibi presented "Globus: Connect Deployment for the H3ABioNet consortium". This software ( https://www.globus.org/) can be used by the H3ABioNet nodes network in order to be able to transfer large datasets between nodes, independently of the bandwidth and quality of their Internet connections. He developed standard operating procedures for the configuration of hosts supporting Globus and for the installation of the software, and outlined best practices for using it, either for individual users or at the institutional level. So far, a large amount of biological data transfer has occurred between some nodes (IGB-USA, CBIO-South Africa), and from those tests we have gathered useful data about future transfer between those nodes ^6^.

Olasehinde Grace gave a presentation about “Reduction in Prevalence of Plasmodium falciparum Chloroquine/Amodiaquine Resistance (Pfcrt) and Multidrug Resistance (Pfmdr I) genes in Southwestern Nigeria”. In this study, the combination of Pfcrt and Pfmdr1 mutations in isolates associated with chloroquine and amodiaquin resistance was observed in Southwestern Nigeria. His result showed that out of the 140 Plasmodium falciparum-positive samples, 5.7% harbored the chloroquine/amodiaquine-resistant gene (Pfcrt) while 7.1% harbored the multidrug-resistant gene (Pfmdr 1) ^7^.

Raphael Z Sangeda presented the “Data migration of consistent Sickle Cell Disease clinical-database at Muhimbili National Hospital in Tanzania”. The main objective was to migrate the old Sickle cell disease (SCD) database into a new consistent database of patients attending the clinic at Muhimbili National Hospital, in Dar es Salaam, Tanzania. The new database was modeled using MySQL-Workbench by consulting the study case report forms (CRF) and the old database. Consistency, range check and mapping were done using python scripts or VBA scripts. The newly created repository will facilitate analysing the clinical data and generate new interventions for SCD ^8^.

Trust Odia who introduced “Predicting the structure of Anopheles gambiae, cytochrome P450 protein *In-silico*”, delivered the last oral presentation. This work employs *in-silico* methods to predict the structure of the metabolic catalyzer and further deduced a specific function for the same protein. He used the GOPET webtool to deduce the unique function of this protein. He was able to identify and analyze the folds and found the proteins to be active in binding of molecules with an 86% confidence value with various catalysis activities. 20 helices, 16 strands, 48 turn and 348 hydrogen bonds were elucidated and analyzed on the structure ^9^.

### Award winners

H3AbioNet awarded 15 partial or full students travel fellowships to attend the SCS-Africa. In addition, the Mohamed Vth University of Rabat, Morocco supported two travel fellowships.

Based on delegates’ votes, Mohamed Alibi was selected to receive the best oral presentation award, generously supported by Oxford University Press journal Bioinformatics. Alibi’s presentation was about his work entitled “Globus Connect Deployment for the H3ABioNet consortium” ^6^.

## Conclusion

We believe that such an event can prove to be a useful platform for both introducing attendees to the research of other students and young researchers around Africa and also to improve contacts and collaborations among them.
